# What’s normal? Oligosaccharide concentrations and profiles in milk produced by healthy women vary geographically^1^,^2^

**DOI:** 10.3945/ajcn.116.139980

**Published:** 2017-03-29

**Authors:** Michelle K McGuire, Courtney L Meehan, Mark A McGuire, Janet E Williams, James Foster, Daniel W Sellen, Elizabeth W Kamau-Mbuthia, Egidioh W Kamundia, Samwel Mbugua, Sophie E Moore, Andrew M Prentice, Linda J Kvist, Gloria E Otoo, Sarah L Brooker, William J Price, Bahman Shafii, Caitlyn Placek, Kimberly A Lackey, Bianca Robertson, Susana Manzano, Lorena Ruíz, Juan M Rodríguez, Rossina G Pareja, Lars Bode

**Affiliations:** 3 School of Biological Sciences,; 4 Paul G Allen School for Global Animal Health, and; 5 Department of Anthropology, Washington State University, Pullman, WA;; 6 Department of Animal and Veterinary Science,; 7 Program in Bioinformatics and Computational Biology,; 8 Department of Biological Sciences, and; 9 Statistical Programs, College of Agricultural and Life Sciences, University of Idaho, Moscow, ID;; 10 Dalla Lana School of Public Health, University of Toronto, Toronto, Canada;; 11 Department of Human Nutrition, Egerton University, Nakuru, Kenya;; 12 Medical Research Council (MRC), Human Nutrition Research, Elsie Widdowson Laboratory, Cambridge, United Kingdom;; 13 MRC Unit, Banjul, The Gambia;; 14 MRC International Nutrition Group, London School of Hygiene and Tropical Medicine, London, United Kingdom;; 15 Faculty of Medicine, Lund University, Lund, Sweden;; 16 Department of Nutrition and Food Science, University of Ghana, Accra, Ghana;; 17 Department of Pediatrics and; 18 Mother Milk Infant Center of Research Excellence, University of California, San Diego, La Jolla, CA;; 19 Department of Nutrition, Food Science, and Food Technology, Complutense University of Madrid, Madrid, Spain; and; 20 Nutrition Research Institute, Lima, Peru

**Keywords:** breastfeeding, carbohydrates, human milk, lactation, oligosaccharides

## Abstract

**Background:** Human milk is a complex fluid comprised of myriad substances, with one of the most abundant substances being a group of complex carbohydrates referred to as human milk oligosaccharides (HMOs). There has been some evidence that HMO profiles differ in populations, but few studies have rigorously explored this variability.

**Objectives:** We tested the hypothesis that HMO profiles differ in diverse populations of healthy women. Next, we examined relations between HMO and maternal anthropometric and reproductive indexes and indirectly examined whether differences were likely related to genetic or environmental variations.

**Design:** In this cross-sectional, observational study, milk was collected from a total of 410 healthy, breastfeeding women in 11 international cohorts and analyzed for HMOs by using high-performance liquid chromatography.

**Results:** There was an effect of the cohort (*P* < 0.05) on concentrations of almost all HMOs. For instance, the mean 3-fucosyllactose concentration was >4 times higher in milk collected in Sweden than in milk collected in rural Gambia (mean ± SEM: 473 ± 55 compared with 103 ± 16 nmol/mL, respectively; *P* < 0.05), and disialyllacto-*N*-tetraose (DSLNT) concentrations ranged from 216 ± 14 nmol/mL (in Sweden) to 870 ± 68 nmol/mL (in rural Gambia) (*P* < 0.05). Maternal age, time postpartum, weight, and body mass index were all correlated with several HMOs, and multiple differences in HMOs [e.g., lacto-*N*-neotetrose and DSLNT] were shown between ethnically similar (and likely genetically similar) populations who were living in different locations, which suggests that the environment may play a role in regulating the synthesis of HMOs.

**Conclusions:** The results of this study support our hypothesis that normal HMO concentrations and profiles vary geographically, even in healthy women. Targeted genomic analyses are required to determine whether these differences are due at least in part to genetic variation. A careful examination of sociocultural, behavioral, and environmental factors is needed to determine their roles in this regard. This study was registered at clinicaltrials.gov as NCT02670278.

See corresponding article on page 1027.

## INTRODUCTION

Human milk oligosaccharides (HMOs)^23^ are complex glycans that are highly abundant in human milk (1). Mature human milk contains from 5 to 20 g HMOs/L, which often exceed concentrations of protein (2–7); concentrations are even higher in colostrum (2, 8). In contrast, bovine milk contains lesser amounts of oligosaccharides, and their structures differ greatly from those in human milk (9–12). Decades of research have suggested that HMOs may be important for nourishing health-promoting bacteria in the breastfed infant’s gastrointestinal tract (13–17), and emerging research suggests that HMOs act as antiadhesives, thereby reducing pathogen attachment and infectivity (18–22). HMOs also appear to act as antimicrobials that prevent pathogen proliferation (23) and as epithelial and immune cell modulators that affect host responses (24–26). HMOs may even be involved in brain development (27). As such, an understanding of HMO origins and functions, many of which are structure specific (28), as well as variations in intake by infants may lend key insights into the optimization of infant health and wellbeing during this critical phase of the life cycle.

Although there are substantial variations in HMO concentrations and profiles in women (29), very little is known about the basis of this variability aside from the activity of galactoside 2-α-l-fucosyltransferase 2 (*FUT2*) and galactoside 3/4-l-fucosyltransferase (*FUT3*) genes, which influence the presence or absence of α1-2-fucosylated and α1-3/4-fucosylated HMOs, respectively, as well as many other HMO structures (30, 31). Perhaps the most relevant study to date in which the interpopulation variation in HMO profiles was investigated was conducted by Erney et al. (32), who compared neutral oligosaccharides in milk that were collected from 435 women who were living in 10 countries. Although the authors recognized that, in some cases, small sample sizes within a country made comparisons and generalizations difficult, several findings were of significance. For instance, 2′-fucosyllactose was quantifiable in every milk sample that was collected in Mexico (*n* = 156) and Sweden (*n* = 7), but in only 46% of samples that were collected in the Philippines (*n* = 22). Sweden presented a particularly interesting picture with all samples containing 8 of 9 HMOs studied; none of the samples contained 3-fucosyllactose.

The primary objective of this study was to expand on the work of Erney et al. (32) to reexamine, with the use of more-advanced methods and rigorous sampling approaches, the hypothesis that HMO concentrations and profiles differ in diverse populations. As our secondary objective, we explored relations between selected maternal variables and HMO concentrations; in a subset of samples, we also indirectly examined whether differences in HMOs were more likely related to genetic or environmental factors.

## METHODS

### Experimental design, subjects, and ethics approvals

This investigation took place between May 2014 and April 2016 and was carried out as a cross-sectional, epidemiologic cohort study that involved multiple international sites. To be eligible for participation, women had to be breastfeeding or pumping ≥5 times/d (to ensure adequate milk production), have self-reported having healthy and nursing healthy infants, be ≥18 y of age, and be between 2 wk and 5 mo postpartum. Women did not need to be exclusively breastfeeding. Exclusion criteria included a current indication of a breast infection or breast pain that the woman did not consider normal for lactation, the maternal use of antibiotics in the previous 30 d, or the nursing of a child with signs or symptoms of an acute illness in the previous 7 d or having taken antibiotics in the previous 30 d.

Our sample included 2 European (Spanish and Swedish), 1 South American (Peruvian), 2 North American, and 6 sub-Saharan African (rural and urban Ethiopian, rural and urban Gambian, Ghanaian, and Kenyan) populations and cohorts. Spanish subjects were recruited in Madrid, Zaragoza, Huesca, and Vizcaya with no additional requirements in terms of ethnicity. Swedish subjects were recruited in or near Helsingborg and had self-reported as Nordic (both parents and all grandparents were self-described as having only Swedish, Finnish, Danish, Icelandic, or Norwegian heritage). Peruvian subjects resided in a peri-urban area of Lima. North American subjects were recruited in Southeastern Washington and Northwestern Idaho [United States–Washington (US_W_)] and Southern California [United States–California (Hispanic) (US_C_)]; the former group was of unspecified ethnicity, and the latter group was self-identified as Hispanic. Both rural and urban Ethiopian subjects were self-identified as Sidama and were assumed to be genetically similar. Rural Ethiopian participants resided in the highlands of the Southern Nations, Nationalities, and Peoples’ Region, whereas urban participants resided in Hawassa, which is also in the Southern Nations, Nationalities, and Peoples’ Region. Rural and urban Gambian subjects had self-identified as Mandinka and were assumed to be genetically similar. Urban Gambian participants resided in the Bakau region, whereas the rural cohort stemmed from the West Kiang region. Ghanaian subjects were Krobo or Dangme and lived in southeastern Ghana. Kenyan subjects were recruited from the multiethnic city of Nakuru. Our goal was to obtain data and human milk samples from 40 women in each cohort, which was a number that was primarily chosen to fit within the available resources and time.

On enrollment, each woman completed several questionnaires including one questionnaire that ensured eligibility and another questionnaire that was related to general maternal and infant health and anthropometric measures. Ethics approvals were obtained for all procedures from each participating institution and with overarching approval from the Washington State University Institutional Review Board (13264). After being translated from English (when needed), informed, verbal, or written consent (depending on the locale and the subject’s literacy level) was acquired from each participating woman.

### Milk collection and preservation

With the use of gloved hands, research personnel or the mother (depending on cultural acceptability) cleaned the study breast (chosen by the subject) twice with the use of prepackaged castile soap towelettes (Professional Disposables International Inc.) and with a newly opened package each time. When deemed appropriate, this step was preceded by a general cleansing with water (and soap if needed) to remove noticeable soil. In the cohorts in Peru, Sweden, US_C_, and US_W_, ≤200-mL (typically 40–60-mL) milk samples were collected into a single-use, sterile, polypropylene milk-collection container with a polybutylene terephthalate cap (Medela Inc.) with the use of an electric breast pump. In Spain, milk samples were collected via manual expression (with the use of a gloved hand) into single-use, sterile, polypropylene milk-collection containers with polybutylene terephthalate caps (Medela Inc.). At the remaining sites, milk was manually expressed (with the use of a gloved hand) into sterile, polypropylene specimen containers with polyethylene caps (VWR International LLC.). When necessary to collect the desired volume or because the mother requested to switch breasts, milk was expressed from both breasts; when this occurred, the previously detailed methods were repeated with the other breast. To help control for known and unknown biases that might have been introduced through the use of different materials, all milk-collection supplies (e.g., gloves, wipes, and collection containers) were standardized and provided to study personnel at each site.

In all sites except rural Ethiopia (ET_R_) and Peru, milk was immediately placed in ice or in a cold box (4°C) where it remained until it was partitioned, within 1 h, into aliquots. Milk was frozen (−20°C), shipped on dry ice (if necessary; −78.5°C), and again frozen (−20°C) until it was analyzed. In Peru, milk was immediately partitioned into aliquots and frozen (−20°C), shipped on dry ice, and again frozen (−20°C) until it was analyzed. Because the ET_R_ site did not have consistent access to electricity, milk that was collected in this cohort was preserved with a milk-preservation solution (one-to-one ratio) that was contained in a Milk DNA Preservation and Isolation Kit (Norgen Biotek Corp.); this preserved milk was stored at an ambient temperature for ≤1 wk after which it was transferred to a freezer (−20°C), shipped on dry ice, and again frozen (−20°C) until it was analyzed. Unpublished data from our research group confirmed that the use of this preservation method did not influence the HMO analysis (L Bode, MK McGuire, June 2016).

### Oligosaccharide analysis

HPLC was used to characterize HMO in breast milk as previously described (33). Briefly, human milk (20 μL) was spiked with raffinose (a non-HMO carbohydrate) as an internal standard to allow for absolute quantification. Oligosaccharides were extracted with the use of high-throughput solid-phase extraction over C18 and carbograph microcolumns (Thermo Scientific HyperSep) and fluorescently labeled with 2-aminobenzamide. Labeled oligosaccharides were analyzed with the use of HPLC on an amide-80 column with an ammonium formate–acetonitrile buffer system at a concentration of 50-mmol/L. Separation was performed at 25°C and was monitored with the use of a fluorescence detector at a 360-nm excitation and 425-nm emission. The peak annotation was based on standard retention times and a mass spectrometric analysis with the use of a duo ion-trap mass spectrometer (Thermo LCQ) that was equipped with a nano-electrospray ionization source. Absolute concentrations were calculated on the basis of standard response curves for each of the annotated HMOs. The following 19 HMOs were identified and quantified: 2′-fucosyllactose, 3-fucosyllactose, 3′-sialyllactose, 6′-sialyllactose, difucosyllactose, difucosyllacto-*N*-hexaose, difucosyllacto-*N*-tetrose (DFLNT), disialyllacto-*N*-hexaose (DSLNH), disialyllacto-*N*-tetraose (DSLNT), fucodisialyllacto-*N*-hexaose (FDSLNH), fucosyllacto-*N*-hexaose (FLNH), lacto-*N*-fucopentaose (LNFP) I, LNFP II, LNFP III, lacto-*N*-hexaose, lacto-*N*-neotetraose (LNnT), lacto-*N*-tetrose (LNT), sialyl-lacto-*N*-tetraose b (LSTb), and sialyl-lacto-*N*-tetraose c (LSTc). HMOs were also grouped according to common structural elements. Secretor milk was defined as having a 2′-fucosyllactose concentration that was greater than a natural, very low break in the data. The total concentration of HMOs was calculated as the sum of the annotated oligosaccharides. The proportion of each HMO that made up the total HMO concentration was also calculated. HMO concentrations were analyzed with the use of both a molar-based unit of measure (nanomoles per milliliter) and a weight-based unit of measure (micrograms per milliliter). However, in the interest of space and coherence, only the molar data are presented and discussed in this article. Data that were analyzed on a weight basis (micrograms per milliliter) are shown in **Supplemental Tables 1–9**.

### Statistical analysis

All exploratory and descriptive statistical analyses were performed with the use of R software (version 3.3.2; R Foundation for Statistical Computing) (34). To correct for nonnormal (right-skewness) distributions, HMO quantities were log transformed before analyses. The effect of the cohort on total, individual, and grouped HMO concentrations was tested via 1-factor ANOVA procedures with the use of the AOV option in the stats package in R software. Multiple comparisons were carried out with the use of Bonferroni adjustment [LSD.test in the agricolae package (35)] to assess differences in populations. Differences in proportions of each cohort that were characterized as being secretors were tested with the use of a chi-square post hoc procedure in the NCStats package (36) with Benjamini and Hochberg false-discovery-rate corrections (37). α-Diversity metrics including richness, the Shannon diversity index, the inverse Simpson index, Shannon evenness, Simpson evenness, and Pielou evenness were computed (38). The AOV procedure was also used to examine the effect of the cohort on richness, evenness, and diversity indexes and to examine the effect of the cohort on selected metadata [maternal age, parity, time postpartum, and BMI (in kg/m^2^)].

To visualize and characterize associations between individual HMO or HMO profiles and selected metadata, heat maps of Spearman-rank correlation coefficients were constructed with the use of the corrplot package (39). To help control for the many correlations in which we were interested while also wanting to fully explore the many relations that might have been of interest in this exploratory component of our data analysis, associations were deemed significant with the assumption of α = 0.01.

Multivariate analyses to explore patterns and similarities in complex HMO profiles were followed and included nonmetric multidimensional scaling analyses with the use of a Bray-Curtis dissimilarity matrix [metaMDS procedure in the vegan package (38) and ggplot2 package (40) and a principle components analysis princomp procedure in the stats base package of R software]. Within these analyses, potential groupings of HMO profiles by cohort, continent and ethnicity, BMI, time postpartum, parity, and maternal age were examined. In this evaluation, continuous variables were categorized as follows: BMI (<18.5, 18.5–24.9, and ≥25); time postpartum (quartiles: 20–46, 47–63, 64–78, and 79–161 d); parity (1, 2, and ≥3 children); and maternal age (quartiles: 18–22, 23–27, 28–32, and 33–46 y). Nonnegative matrix factorization (NMF) was also used to discern potential patterns in the HMO profile data (41). In this set of analyses, data were processed with the use of the Brunet method (42), and 6 basis components were retained on the basis of the rank estimate that was determined from the same package. Heat maps of the NMF feature scores were created with the heatmap.2 procedure in the gplots package (43) to look for patterns within the data structure (distinct from the correlation maps and shown in **Supplemental Figures 1** and **2**).

## RESULTS

### Description of participating women

A total of 413 women were enrolled; 41, 40, 40, 40, 42, 42, 43, 41, 24, 41, and 19 women were from ET_R_, urban Ethiopia (ET_U_), rural Gambia (GB_R_), urban Gambia (GB_U_), Ghana, Kenya, Peru, Spain, Sweden, US_W_, and US_C_, respectively. Milk samples from all of these women, except for 2 women in Ghana and 1 woman in ET_R_, were successfully obtained and analyzed. Consequently, data from a total of 410 women were included in our analysis. Basic anthropometric and demographic information of these participants is shown in **Table 1**. Several of these classifications differed in the cohorts. For example, women in ET_U_ were younger than all other groups except for their counterparts in ET_R_, GB_R_, Kenya, and Peru. Parity in women in ET_R_ and GB_R_ was higher than that of women in ET_U_, Spain, Sweden, and US_W_. Body weight also varied greatly in the cohorts whereby women in Peru, Sweden, US_C_, US_W_, Spain, Ghana, and GB_U_ were relatively heavier and had higher BMI, and women in ET and GB_R_ were lighter and had lower BMI. Note that groups in ET_R_ than ET_U_ as well as cohorts in GB_R_ than GB_U_ were, for the most part, similar in terms of these variables although parity was higher in women in ET_R_ than in ET_U_; there were no differences in these factors between the 2 US cohorts.

**TABLE 1:** Characteristics of women (*n* = 410) participating in the study and effects of population^1^

	Ethiopia		Gambia							United States	
Variable	Rural (*n* = 40)	Urban (*n* = 40)	Rural (*n* = 40)	Urban (*n* = 40)	Ghana (*n* = 40)	Kenya (*n* = 42)	Peru (*n* = 43)	Spain (*n* = 41)	Sweden (*n* = 24)	Washington (*n* = 41)	California (*n* = 19)
Age,^2^ y	24.6 ± 0.8^c,d^	21.7 ± 0.5^d^	26.9 ± 1.2^b–d^	27.0 ± 0.8^b,c^	28.9 ± 0.9^a–c^	25.4 ± 0.8^b–d^	26.7 ± 1.0^b–d^	34.3 ± 0.6^a^	30.9 ± 1.1^a,b^	29.0 ± 0.8^a–c^	29.0 ± 1.1^a–c^
Parity, *n*	3.6 ± 0.3^a^	1.7 ± 0.2^c,d^	4.2 ± 0.5^a^	3.3 ± 0.3^a,b^	2.3 ± 0.2^a–d^	2.5 ± 0.2^a–c^	2.0 ± 0.1^a–d^	1.3 ± 0.1^d^	1.6 ± 0.2^b–d^	1.8 ± 0.2^b–d^	1.8 ± 0.2^a–d^
Time postpartum,^3^ d	71 ± 5	59 ± 2	65 ± 3	62 ± 3	58 ± 3	73 ± 4	60 ± 3	70 ± 4	49 ± 4	68 ± 3	62 ± 5
Weight,^4^ kg	51.0 ± 1.3^c^	55.6 ± 1.2^b,c^	56.3 ± 1.3^b,c^	64.2 ± 1.7^a,b^	63.3 ± 1.7^a,b^	60.1 ± 1.5^b,c^	65.5 ± 2.0^a,b^	64.3 ± 1.5^a,b^	73.6 ± 2.6^a^	75.0 ± 2.4^a^	76.3 ± 3.0^a^
Height,^5^ cm	155 ± 1^d,e^	159 ± 1^c,d^	162 ± 1^a–c^	167 ± 1^a^	159 ± 1^c,d^	159 ± 1^b–d^	153 ± 1^e^	165 ± 1^a,b^	169 ± 1^a^	167 ± 1^a^	162 ± 1^a–d^
BMI,^5^ kg/m^2^	21.3 ± 0.4^d^	22.1 ± 0.5^c,d^	21.4 ± 0.5^c,d^	23.0 ± 0.6^b–d^	25.0 ± 0.6^a–d^	23.6 ± 0.6^b–d^	28.1 ± 0.8^a^	23.5 ± 0.6^b–d^	25.8 ± 1.0^a–c^	26.8 ± 0.8^a,b^	29.1 ± 1.1^a^

1 All values are means ± SEMs. Values in a row that do not share a common superscript letter differed with the use of Bonferroni-correction procedures for multiple comparisons, *P* < 0.05. All statistical inferences were based on log-transformed data.

2 Because of missing data (although we are confident that all women were of the appropriate age range), *n* = 38 and 39 for rural and urban Gambia cohorts, respectively.

3 Because of missing data, *n* = 39 for the rural Gambia cohort.

4 Because of missing data, *n* = 39, 41, and 37 in rural Gambia, Kenya, and United States–Washington cohorts, respectively.

5 Because of missing data, *n* = 40 in the Kenya cohort.

### Effects of cohort on individual HMO concentrations and HMO groupings

**FIGURE 1:**
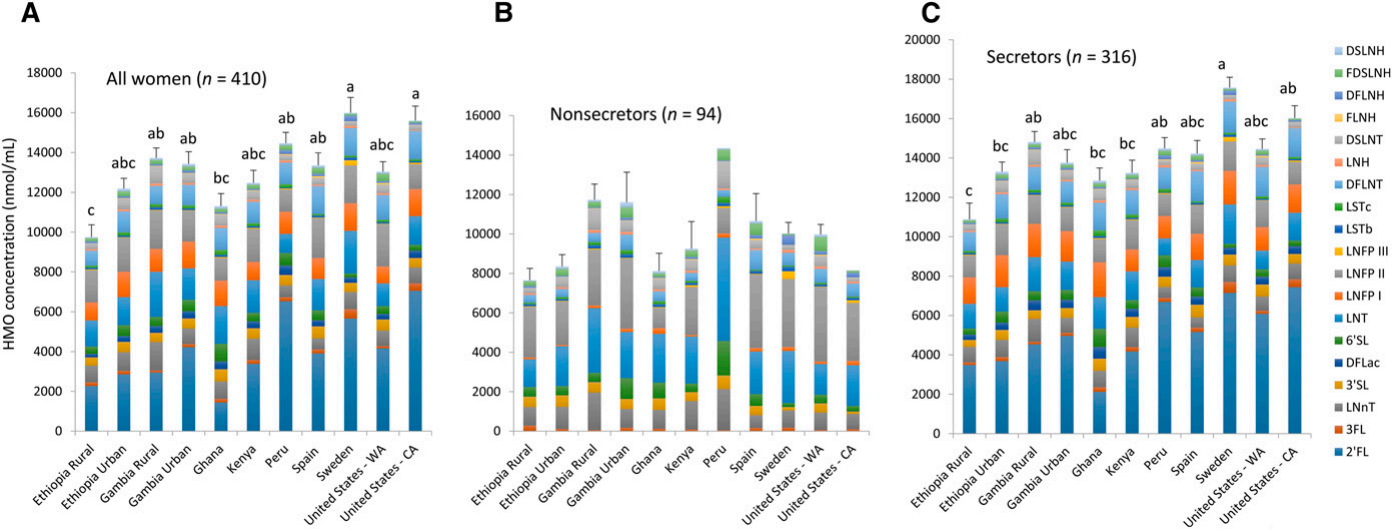
Mean ± SEM absolute total and HMO isoform concentrations of all women combined (A), nonsecretors (B), and secretors (C). (A and B) Bars without a common lowercase letter represent total HMO values that differed with the use of Bonferroni-correction procedures for multiple comparisons. All statistical inferences were carried out on log-transformed data. Note that there was only one nonsecretor subject each in Peru and United States - CA. CA, California; DFLac, difucosyllactose; DFLNH, difucosyllacto-*N*-hexaose; DFLNT, difucosyllacto-*N*-tetrose; DSLNH, disialyllacto-*N*-hexaose; DSLNT, disialyllacto-*N*-tetraose; FDSLNH, fucodisialyllacto-*N*-hexaose; FLNH, fucosyllacto-*N*-hexaose; HMO, human milk oligosaccharide; LNFP, lacto-*N*-fucopentaose; LNH, lacto-*N*-hexaose; LNnT, lacto-*N*-neotetraose; LNT, lacto-*N*-tetrose; LSTb, sialyl-lacto-*N*-tetraose b; LSTc, sialyl-lacto-*N*-tetraose c; WA, Washington; 2′FL, 2′-fucosyllactose; 3FL, 3-fucosyllactose; 3′SL, 3′-sialyllactose; 6′SL, 6′-sialyllactose.

**FIGURE 2:**
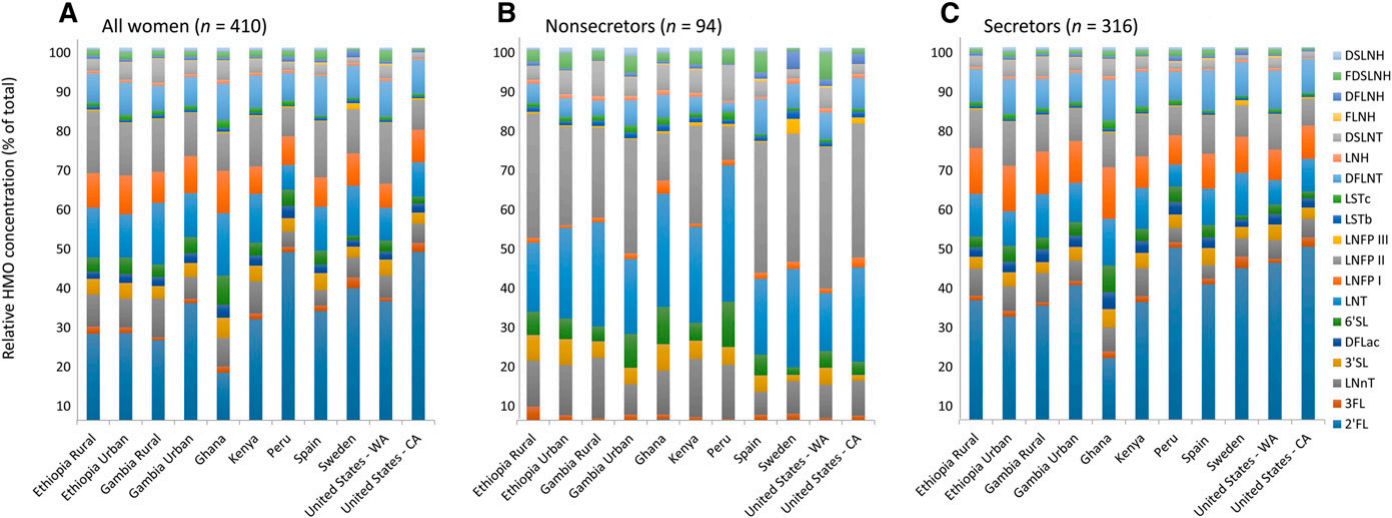
Mean ± SEM relative abundance of HMO concentrations of all women combined (A), nonsecretors (B), and secretors (C) in each cohort. Note that there was only one nonsecretor subject each in Peru and United States - CA. CA, California; DFLac, difucosyllactose; DFLNH, difucosyllacto-*N*-hexaose; DFLNT, difucosyllacto-*N*-tetrose; DSLNH, disialyllacto-*N*-hexaose; DSLNT, disialyllacto-*N*-tetraose; FDSLNH, fucodisialyllacto-*N*-hexaose; FLNH, fucosyllacto-*N*-hexaose; HMO, human milk oligosaccharide; LNFP, lacto-*N*-fucopentaose; LNH, lacto-*N*-hexaose; LNnT, lacto-*N*-neotetraose; LNT, lacto-*N*-tetrose; LSTb, sialyl-lacto-*N*-tetraose b; LSTc, sialyl-lacto-*N*-tetraose c; WA, Washington; 2′FL, 2′-fucosyllactose; 3FL, 3-fucosyllactose; 3′SL, 3′-sialyllactose; 6′SL, 6′-sialyllactose.

Mean values for individual and total HMO concentrations for each cohort are provided in **Table 2** (all women) and visually depicted in **Figure 1** (all women, secretors, and nonsecretors). Relative abundances of each HMO in all women, secretors, and nonsecretors in each cohort are shown in **Figure 2**. There was an effect of the cohort on the total HMO concentration and the concentrations of all the HMO types except for LNFP I. For instance, DSLNT concentrations ranged from a low of 216 ± 14 nmol/mL in Sweden to a high of 870 ± 68 nmol/mL in GB_R_ (*P* < 0.05). LNFP III was significantly higher in milk that was produced by Swedish women than by all other cohorts (*P* < 0.05) except for women in the US_C_; and LSTb was lower (*P* < 0.05) in milk that was produced by women in Peru and the US_C_ than by all other cohorts. In addition, although they did not reach significance with the use of Bonferroni correction for multiple comparisons, 2′-fucosyllactose concentrations were 4–5 times higher in samples that were collected in the US_C_ (7043 ± 858 nmol/L) and Peru (6528 ± 435 nmol/L) than in those that were collected in Ghana (1428 ± 207 nmol/mL).

**TABLE 2:** Variation in total and individual HMOs in 410 healthy women living in selected locations around the world^1^

	Ethiopia		Gambia							United States	
HMO	Rural (*n* = 40)	Urban (*n* = 40)	Rural (*n* = 40)	Urban (*n* = 40)	Ghana (*n* = 40)	Kenya (*n* = 42)	Peru (*n* = 43)	Spain (*n* = 41)	Sweden (*n* = 24)	Washington (*n* = 41)	California (*n* = 19)
2′FL, nmol/mL	2264 ± 370	2853 ± 369	2950 ± 455	4220 ± 530	1438 ± 207	3380 ± 422	6528 ± 435	3906 ± 464	5661 ± 728	4159 ± 531	7043 ± 858
3FL, nmol/mL	189 ± 22^a,b,c^	184 ± 42^b,c^	103 ± 16^c^	162 ± 21^b,c^	192 ± 33^b,c^	195 ± 28^b,c^	209 ± 32^a,b,c^	206 ± 25^a,b,c^	473 ± 55^a^	122 ± 14^b,c^	388 ± 47^a,b^
LNnT, nmol/mL	838 ± 60^a,b,c^	927 ± 67^a,b,c^	1423 ± 117^a^	781 ± 61^b,c^	866 ± 70^a,b,c^	1073 ± 103^a,b^	588 ± 56^c^	548 ± 45^c^	854 ± 74^a,b,c^	776 ± 42^b,c^	793 ± 82^a,b,c^
3′SL, nmol/mL	413 ± 33	526 ± 43	465 ± 36	505 ± 45	618 ± 56	528 ± 44	528 ± 51	607 ± 42	467 ± 65	562 ± 40	473 ± 55
DFLac, nmol/mL	179 ± 27	290 ± 33	338 ± 59	355 ± 42	393 ± 65	338 ± 38	470 ± 49	307 ± 40	275 ± 40	270 ± 38	374 ± 57
6′SL, nmol/mL	374 ± 63^c,d^	545 ± 45^a,b^	462 ± 35^a,b,c^	585 ± 61^a,b^	890 ± 88^a^	435 ± 34^b,c^	636 ± 63^a,b^	504 ± 40^a,b,c^	200 ± 24^d^	402 ± 34^b,c,d^	294 ± 49^c,d^
LNT, nmol/mL	1304 ± 131^b,c^	1408 ± 113^a,b^	2265 ± 222^a^	1576 ± 143^a^	1882 ± 209^a^	1632 ± 158^a^	953 ± 139^b^	1570 ± 119^a^	2132 ± 210^a^	1135 ± 91^a,b^	1438 ± 198^a,b^
LNFP I, nmol/mL	904 ± 167	1276 ± 205	1153 ± 208	1343 ± 193	1292 ± 224	921 ± 158	1116 ± 112	1056 ± 167	1395 ± 220	850 ± 144	1368 ± 143
LNFP II, nmol/mL	1618 ± 165^b^	1713 ± 151^a,b^	1925 ± 201^a,b^	1551 ± 190^a,b^	1133 ± 89^a,b^	1667 ± 167^b^	1115 ± 105^a,b^	2001 ± 227^a,b^	1893 ± 205^a,b^	2125 ± 216^a,b^	1240 ± 154^a^
LNFP III, nmol/mL	44 ± 8^b,c^	24 ± 3^c^	40 ± 7^b,c^	30 ± 4^b,c^	47 ± 7^b,c^	46 ± 10^b,c^	53 ± 8^b,c^	32 ± 4^b,c^	269 ± 22^a^	25 ± 5^c^	76 ± 10^a,b^
LSTb, nmol/mL	86 ± 7^a^	79 ± 7^a^	132 ± 10^a^	96 ± 12^a^	115 ± 10^a^	86 ± 1^a^	41 ± 5^b^	105 ± 15^a^	140 ± 15^a^	82 ± 7^a^	79 ± 14^a,b^
LSTc, nmol/mL	101 ± 19^c^	169 ± 15^a,b^	159 ± 13^a,b^	146 ± 20^a,b,c^	246 ± 25^a^	158 ± 22^a,b,c^	182 ± 17^a,b^	72 ± 7^c^	92 ± 16^b,c^	112 ± 12^b,c^	103 ± 12^b,c^
DFLNT, nmol/mL	758 ± 127	1057 ± 147	913 ± 135	1032 ± 139	1105 ± 188	1082 ± 134	1076 ± 95	1406 ± 120	1388 ± 147	1246 ± 105	1418 ± 197
LNH, nmol/mL	68 ± 8	86 ± 9	112 ± 17	92 ± 15	109 ± 13	92 ± 10	107 ± 10	58 ± 4	113 ± 12	93 ± 7	39 ± 5
DSLNT, nmol/mL	310 ± 30^c,d^	553 ± 42^a,b^	870 ± 68^a^	477 ± 45^b,c^	561 ± 57^a,b^	444 ± 47^b,c,d^	274 ± 33^d^	357 ± 31^b,c,d^	216 ± 14^d^	443 ± 28^b,c,d^	275 ± 14^b,c,d^
FLNH, nmol/mL	5 ± 1^c^	27 ± 5^b^	30 ± 5^a,b^	35 ± 5^a,b^	32 ± 7^b^	33 ± 6^b^	50 ± 7^b,c^	83 ± 9^a^	10 ± 3^b,c^	73 ± 7^a^	7 ± 1^b,c^
DFLNH, nmol/mL	84 ± 15^c^	94 ± 16^c^	87 ± 13^c^	123 ± 18^a,b,c^	81 ± 11^b,c^	64 ± 10^c^	195 ± 21^a,b^	115 ± 17^a,b,c^	285 ± 32^a^	98 ± 12^b,c^	93 ± 11^a,b,c^
FDSLNH, nmol/mL	158 ± 30^a,b^	240 ± 26^a,b^	197 ± 24^a,b^	199 ± 33^a,b^	182 ± 25^a,b^	204 ± 25^a,b^	245 ± 33^a,b^	314 ± 38^a^	83 ± 15^b^	370 ± 48^a^	70 ± 9^b^
DSLNH, nmol/mL	50 ± 7^b^	136 ± 12^a^	109 ± 12^a,b^	129 ± 20^a,b^	126 ± 18^a,b^	101 ± 11^a,b^	108 ± 16^a,b^	103 ± 11^a,b^	55 ± 11^a,b^	93 ± 12^a,b^	33 ± 6^b^
Total, nmol/mL	9748 ± 626^c^	12,187 ± 519^a,b,c^	13,732 ± 497^a,b^	13,435 ± 613^a,b^	11,307 ± 631^b,c^	12,480 ± 628^a,b,c^	14,474 ± 539^a,b^	13,349 ± 645^a,b^	15,998 ± 768^a^	13,035 ± 502^a,b^	15,606 ± 727^a,b^

1 All values are means ± SEMs. Values in a row that do not share a common superscript letter differed with the use of Bonferroni-correction procedures for multiple comparisons, *P* < 0.05. All statistical inferences were based on log-transformed data. DFLac, difucosyllactose; DFLNH, difucosyllacto-*N*-hexaose; DFLNT, difucosyllacto-*N*-tetrose; DSLNH, disialyllacto-*N*-hexaose; DSLNT, disialyllacto-*N*-tetraose; FDSLNH, fucodisialyllacto-*N*-hexaose; FLNH, fucosyllacto-*N*-hexaose; HMO, human milk oligosaccharide; LNFP, lacto-*N*-fucopentaose; LNH, lacto-*N*-hexaose; LNnT, lacto-*N*-neotetraose; LNT, lacto-*N*-tetrose; LSTb, sialyl-lacto-*N*-tetraose b; LSTc, sialyl-lacto-*N*-tetraose c; 2′FL, 2′-fucosyllactose; 3FL, 3-fucosyllactose; 3′SL, 3′-sialyllactose; 6′SL, 6′-sialyllactose.

Several differences were also shown between rural and urban sites in Ethiopia and between rural and urban sites in The Gambia despite the fact that, within each country, the populations studied were expected to have been genetically related. For instance, in The Gambia, the LNnT concentration of milk that was produced by the rural cohort was higher than that produced by the urban cohort (1423 ± 117 compared with 781 ± 61 nmol/mL, respectively; *P* < 0.05). The same difference was shown for DSLNT (870 ± 68 compared with 477 ± 45 nmol/mL, respectively; *P* < 0.05). Conversely, although they were similar between rural and urban Gambian cohorts, concentrations of 6′-sialyllactose, LSTc, and FLNH were higher in milk that was produced by mothers in ET_U_ than by mothers in ET_R._

There were also several differences between the 2 US populations despite the fact that they were very similar in terms of anthropometric and reproductive variables. For instance, FDSLNH was higher in the US_W_ group than in the US_C_ group (370 ± 48 compared with 70 ± 9 nmol/mL, respectively; *P* < 0.05). Because both ethnicity and location, both of which are likely related to environmental variables such as the diet, were different between these groups; however, further work will be required to tease apart potentially causative factors.

Other groupings of HMOs that are based on factors such as HMO-bound sialic acid, chain type, and linkage type also revealed differences in cohorts (**Table 3**). For instance, milk that was produced by women in Sweden and the US_C_ was the most fucosylated and the least sialylated; milk from mother in ET_R_ was less sialylated than that of women in ET_U_; and milk produced by women in Peru was highly enriched with small HMOs (defined as the sum of 2′-fucosyllactose, 3-fucosyllactose, 3′-sialyllactose, and trisaccharides).

**TABLE 3:** Variation in HMO groupings in 410 healthy women living in selected locations around the world^1^

	Ethiopia		Gambia							United States	
Variable	Rural (*n* = 40)	Urban (*n* = 40)	Rural (*n* = 40)	Urban (*n* = 40)	Ghana (*n* = 40)	Kenya (*n* = 42)	Peru (*n* = 43)	Spain (*n* = 41)	Sweden (*n* = 24)	Washington (*n* = 41)	California (*n* = 19)
HMO-bound sialic acid,^2^ nmol/mL	2010 ± 163^b,c^	3179 ± 158^a^	3570 ± 181^a^	2941 ± 212^a^	3605 ± 235^a^	2704 ± 157^a,b^	2640 ± 180^a,b^	2835 ± 150^a^	1606 ± 96^c^	2968 ± 153^a^	1705 ± 106^b,c^
HMO-bound fucose,^3^ nmol/mL	7226 ± 608^b^	9198 ± 621^a,b^	9074 ± 665^a,b^	10,558 ± 614^a,b^	7474 ± 679^b^	9415 ± 659^a,b^	12,797 ± 1090^a^	11,254 ± 640^a,b^	13,679 ± 866^a^	10,953 ± 603^a,b^	13,963 ± 872^a^
Small HMOs,^4^ nmol/mL	3240 ± 357^b^	4108 ± 383^a,b^	3980 ± 466^b^	5471 ± 520^a,b^	3138 ± 266^b^	4538 ± 440^a,b^	7900 ± 441^a^	5223 ± 481^a,b^	6800 ± 813^a,b^	5245 ± 552^a,b^	8199 ± 947^a,b^
Type 1,^5^ nmol/mL	4222 ± 281^b,c^	5029 ± 242^a,b^	6344 ± 345^a^	5043 ± 317^a,b,c^	4984 ± 346^a,b,c^	4751 ± 297^a,b,c^	3499 ± 249^c^	5089 ± 337^a,b,c^	5776 ± 380^a,b^	4634 ± 268^a,b,c^	4400 ± 297^a,b,c^
Type 2,^6^ nmol/mL	983 ± 73b^b,c^	1120 ± 64^a,b^	1622 ± 124^a^	957 ± 69^b,c^	1159 ± 81^a,b^	1277 ± 109^a,b^	824 ± 65^b,c^	652 ± 48^c^	1215 ± 93^a,b^	913 ± 48^b,c^	972 ± 90^a,b,c^
α-1,2,^7^ nmol/mL	3169 ± 520	4129 ± 515	4103 ± 609	5562 ± 690	2731 ± 386	4302 ± 546	7644 ± 505	4962 ± 605	7056 ± 855	5009 ± 634	8412 ± 904
α-1,3,^8^ nmol/mL	232 ± 26^b,c^	208 ± 41^c^	143 ± 16^c^	192 ± 20^c^	239 ± 33^c^	241.2 ± 26.5^b,c^	262 ± 32^b,c^	238 ± 24^b,c^	742 ± 47^a^	147 ± 13^c^	464 ± 44^a,b^
α-2,6,^9^ nmol/mL	561 ± 80^c^	793 ± 58^a,b^	753 ± 46^a,b,c^	828 ± 84^a,b^	1251 ± 110^a^	680 ± 49^b,c^	859 ± 75^a,b^	681 ± 53^b,c^	431 ± 32^c^	595 ± 44^b,c^	476 ± 59^b,c^

1 All values are means ± SEMs. Values in a row that do not share a common superscript letter differed with the use of Bonferroni-correction procedures for multiple comparisons, *P* < 0.05. All statistical inferences were based on log-transformed data. *Overall effect of cohort, *P* < 0.01 (1-factor ANOVA). DFLac, difucosyllactose; DFLNH, difucosyllacto-*N*-hexaose; DFLNT, difucosyllacto-*N*-tetrose; DSLNH, disialyllacto-*N*-hexaose; DSLNT, disialyllacto-*N*-tetraose; FDSLNH, fucodisialyllacto-*N*-hexaose; FLNH, fucosyllacto-*N*-hexaose; HMO, human milk oligosaccharide; LNFP, lacto-*N*-fucopentaose; LNH, lacto-*N*-hexaose; LNnT, lacto-*N*-neotetraose; LNT, lacto-*N*-tetrose; LSTb, sialyl-lacto-*N*-tetraose b; LSTc, sialyl-lacto-*N*-tetraose c; 2′FL, 2′-fucosyllactose; 3FL, 3-fucosyllactose; 3′SL, 3′-sialyllactose; 6′SL, 6′-sialyllactose.

2 Calculated as the sum of all sialic acid moieties bound to each HMO.

3 Calculated as the sum of all fucose moieties bound to each HMO.

4 Calculated as 2′FL + 3FL + 3′SL + 6′SL.

5 Calculated as LNT + LNFP I + LNFP II + LSTb + DSLNT.

6 Calculated as LNnT + LNFP III + LSTc.

7 Calculated as LNFP I + 2′FL.

8 Calculated as LNFP III + 3FL.

9 Calculated as LSTb + LSTc + 6′SL.

### Effects of cohort on secretor status and of secretor status on individual HMO concentrations

**FIGURE 3:**
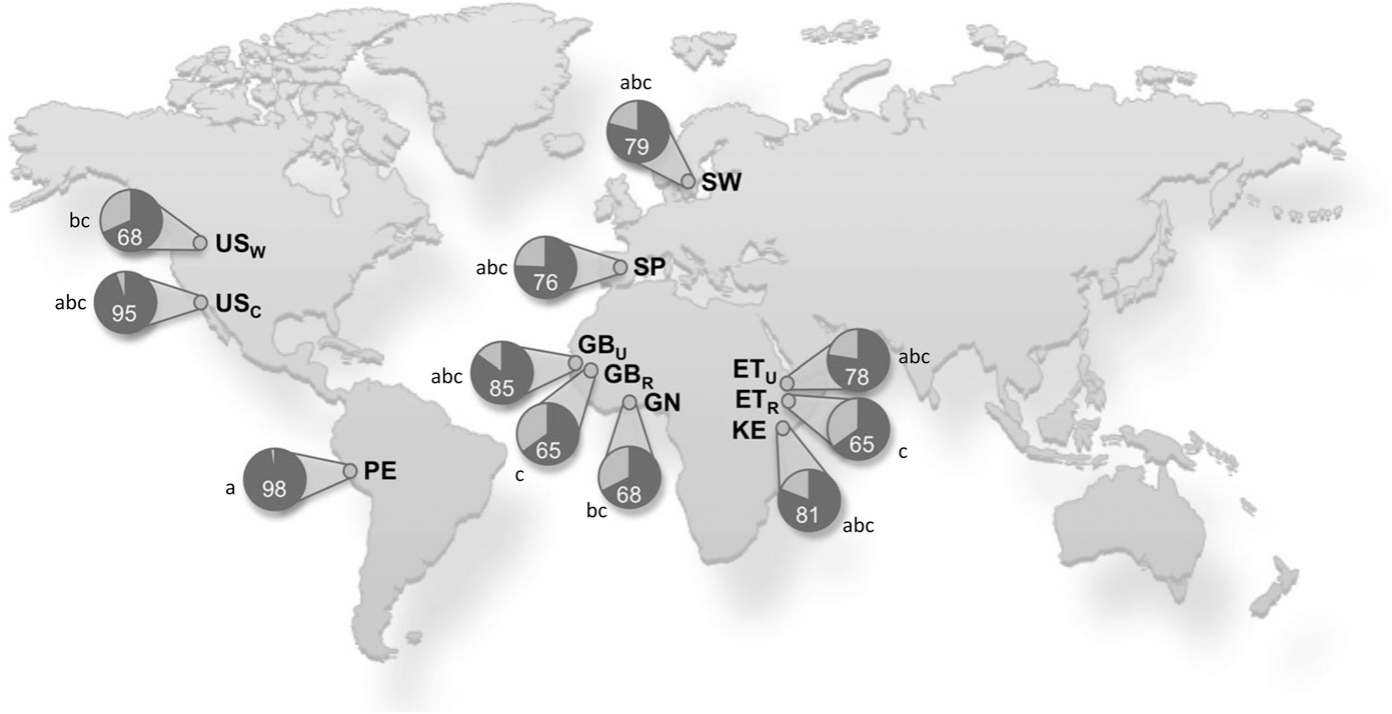
Percentages of women in each cohort categorized as secretors. Cohorts that do not share a common lowercase letter differ (*P* < 0.05) in terms of their percentages of women who were secretors with the use of a chi-square test with Benjamini and Hochberg false-discovery-rate corrections. ET_R_, rural Ethiopia; ET_U_, urban Ethiopia; GB_R_, rural Gambia; GB_U_, urban Gambia; GN, Ghana; KE, Kenya; PE, Peru; SP, Spain; SW, Sweden; US_C_, United States–California (Hispanic); US_W_, United States–Washington.

The proportion of women who were categorized as being secretors (defined as having a 2′-fucosyllactose concentration that was greater than a natural, very low break in the data) was also substantially different in populations (**Figure 3**) and ranged from 65% in populations in GB_R_ and ET_R_ to 98% in the cohort in Peru (*P* < 0.01). The percentage of secretors in the cohort in Peru was also higher than that in the cohorts in Ghana and the US_W_ (98% compared with 68%, respectively; *P <* 0.01) but was similar to that in the cohort in the US_C_ (self-identified as Hispanic) (98% compared with 95%; *P* = 1.00). As anticipated and as illustrated in Figures 1 and , absolute and relative HMO concentrations in secretors and nonsecretors were substantially different (HMO concentrations by secretor status are shown in **Supplemental Tables 10–13**).

### Relations in selected maternal anthropometric, demographic, or reproductive variables and individual HMO concentrations

**FIGURE 4:**
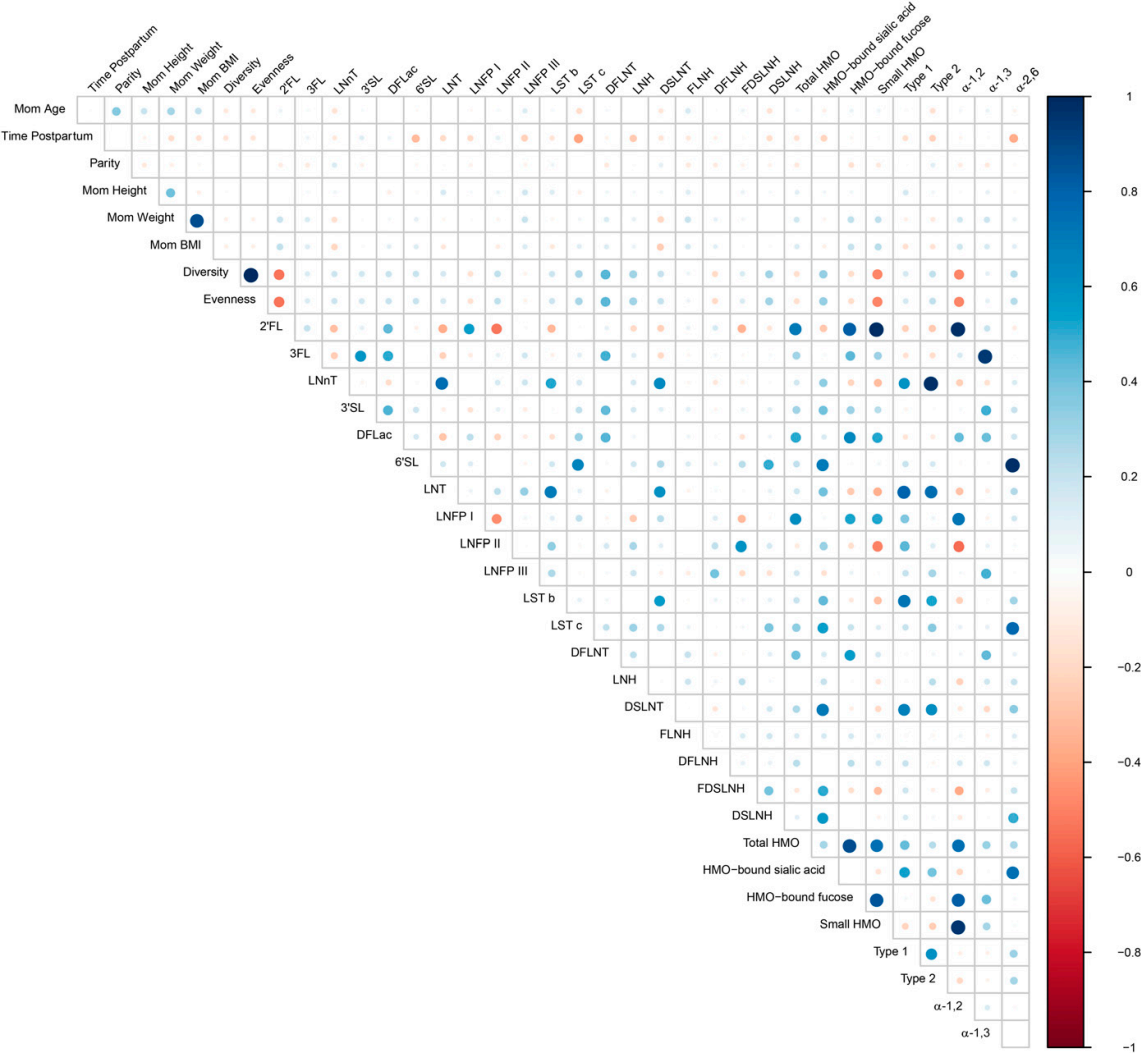
Spearman rank correlations between selected maternal anthropometric, demographic, and reproductive variables and HMO types and groupings. Sizes of dots and colors indicate directionality (blue denotes positive; red denotes negative) and the strength of the association. Total HMO-bound sialic acid; total HMO-bound fucose; small HMO; type 1; type 2; α-1,2; α-1,3; and α-2,6 were calculated as: the sum of all sialic acid moieties bound to each HMO; all fucose moieties bound to each HMO; 2′FL + 3FL + 3′SL + 6′SL; LNT + LNFPI + LNFPII + LSTb + DSLNT; LNnT + LNFPIII + LSTc; LNFP I + 2′FL; LNFP III + 3FL; and LSTb + LSTc + 6′SL, respectively. DFLac, difucosyllactose; DFLNH, difucosyllacto-*N*-hexaose; DFLNT, difucosyllacto-*N*-tetrose; DSLNH, disialyllacto-*N*-hexaose; DSLNT, disialyllacto-*N*-tetraose; FDSLNH, fucodisialyllacto-*N*-hexaose; FLNH, fucosyllacto-*N*-hexaose; HMO, human milk oligosaccharide; LNFP, lacto-*N*-fucopentaose; LNH, lacto-*N*-hexaose; LNnT, lacto-*N*-neotetraose; LNT, lacto-*N*-tetrose; LST, sialyl-lacto-*N*-tetraose; 2′FL, 2′-fucosyllactose; 3FL, 3-fucosyllactose; 3′SL, 3′-sialyllactose; 6′SL, 6′-sialyllactose.

Variations in maternal age, time postpartum, BMI, and weight were associated with several of the HMO types and groups (**Figure 4**). For instance, age was negatively correlated with concentrations of LNnT, LSTc, and DSLNH (*r* = −0.14, −0.17, and −0.15, respectively) and was positively correlated with the concentration of FLNH (*r* = 0.15). Maternal weight and BMI were positively correlated with 2′-fucosyllactose (*r* = 0.20 for both), FLNH (*r* = 0.19 and 0.15, respectively), HMO-bound fucose (*r* = 0.21 for both), and small HMOs (*r* = 0.21 and 0.23, respectively); and maternal weight was positively correlated with LNFP III (*r* = 0.20) and DFLNT (*r* = 0.14). Conversely, maternal weight and BMI were inversely correlated with LNnT and DSLNT (*r* = −0.16 and −0.21, respectively; and *r* = −0.20 and −0.24, respectively). The time postpartum was inversely correlated with several HMOs including 6′-sialyllactose, LNFP III, LSTc, lacto-*N*-hexaose, DSLNT, and *a*2,6-linked oligosaccharides (*r* = −0.31, −0.23, −0.40, −0.26, −0.13, and −0.36, respectively).

### Relations between HMO concentrations

Several correlations also existed in the concentrations of different HMOs and groups thereof. For instance, concentrations of 2′-fucosyllactose, difucosyllactose, and LNFP I were all correlated (*r* = 0.23–0.54); this correlation was expected because their synthesis in the mammary gland is dependent on FUT2 activity. Similarly, concentrations of HMO-bound fucose and α1-2-fucosylated HMO were correlated (*r* = 0.82) as were 2′-fucosyllactose and combined small HMOs (*r* = 0.98). There was also a positive association between LNT and LNnT concentrations (*r* = 0.75). LNFP I and LNFP II were negatively correlated (*r* = −0.46); and 2′-fucosyllactose was negatively correlated with LNFP II (*r* = −0.52). Other associations of interest were that both LNT and LNnT were positively correlated with DSLNT (*r* = 0.60 and 0.62, respectively), and LSTb was positively correlated with DSLNT (*r* = 0.55).

### Effect of cohort on HMO diversity

Diversity metrics also differed in cohorts, and mean values are provided in **Table 4**. In general, HMO diversity and evenness were lowest in milk that was produced by women in Peru and the US_C_ and were highest in milk that was produced by women in Ghana. There were no differences in HMO diversity between rural and urban cohorts in either ET or GB cohorts or between US cohorts (see **Supplemental Tables 14** and **15** for diversity metrics by secretor status).

**TABLE 4:** Variation in HMO diversity and evenness indexes in 410 healthy women living in selected locations around the world^1^

	Ethiopia		Gambia							United States	
Variable	Rural (*n* = 40)	Urban (*n* = 40)	Rural (*n* = 40)	Urban (*n* = 40)	Ghana (*n* = 40)	Kenya (*n* = 42)	Peru (*n* = 43)	Spain (*n* = 41)	Sweden (*n* = 24)	Washington (*n* = 41)	California (*n* = 19)
Shannon entropy	2.06 ± 0.03^a,b,c^	2.15 ± 0.03^a,b^	2.07 ± 0.03^a,b,c^	2.06 ± 0.04^a,b,c^	2.27 ± 0.03^a^	2.10 ± 0.04^a,b,c^	1.94 ± 0.04^b,c^	2.08 ± 0.03^a,b,c^	1.99 ± 0.03^b,c^	2.02 ± 0.03^b,c^	1.86 ± 0.07^c^
Shannon diversity	7.98 ± 0.24^b^	8.79 ± 0.26^a,b^	8.14 ± 0.26^a,b^	8.14 ± 0.31^a,b^	9.84 ± 0.29^a^	8.38 ± 0.27^a,b^	7.25 ± 0.29^b^	8.14 ± 0.20^a,b^	7.40 ± 0.24^b^	7.70 ± 0.24^b^	6.69 ± 0.41^b^
Inverse Simpson	5.51 ± 0.25^b,c^	6.22 ± 0.27^a,b^	5.74 ± 0.23^a,b,c^	5.56 ± 0.29^b,c^	7.31 ± 0.29^a^	5.78 ± 0.26^a,b,c^	4.33 ± 0.25^c^	5.38 ± 0.20^b,c^	4.73 ± 0.25^b,c^	4.92 ± 0.23^b,c^	4.30 ± 0.39^c^
Shannon evenness	0.42 ± 0.01^b^	0.46 ± 0.01^a,b^	0.43 ± 0.01^a,b^	0.43 ± 0.02^a,b^	0.52 ± 0.02^a^	0.44 ± 0.01^a,b^	0.38 ± 0.02^b^	0.43 ± 0.01^a,b^	0.39 ± 0.01^b^	0.41 ± 0.01^b^	0.35 ± 0.02^b^
Simpson evenness	0.29 ± 0.01^b,c^	0.33 ± 0.01^a,b^	0.30 ± 0.01^a,b,c^	0.30 ± 0.02^b,c^	0.38 ± 0.02^a^	0.30 ± 0.01^a,b,c^	0.23 ± 0.01^c^	0.28 ± 0.01^b,c^	0.25 ± 0.01^b,c^	0.26 ± 0.01^b,c^	0.23 ± 0.02^c^
Pielou evenness	0.70 ± 0.01^a,b,c^	0.73 ± 0.01^a,b^	0.70 ± 0.01^a,b,c^	0.70 ± 0.01^a,b,c^	0.77 ± 0.10^a^	0.71 ± 0.01^a,b,c^	0.66 ± 0.01^b,c^	0.71 ± 0.01^a,b,c^	0.67 ± 0.01^b,c^	0.69 ± 0.01^b,c^	0.63 ± 0.02^c^

1 All values are means ± SEMs. Values in a row that do not share a common superscript letter differed with the use of Bonferroni-correction procedures for multiple comparisons, *P* < 0.05. All statistical inferences were based on log-transformed data. HMO, human milk oligosaccharide.

### Effects of cohort and other factors on complex milk oligosaccharide profiles

A visual and numerical evaluation of nonmetric dimensional scaling and principle components analyses and plots (color coded by cohort, continent and ethnicity, BMI, time postpartum, parity, and maternal age) provided no discernible evidence that these factors accounted for an appreciable variability in the overall HMO profiles (MK McGuire, SL Brooker, WJ Price, B Shafii, unpublished results, June 2016). To account for the multivariate nature of the data, the NMF method was considered (43). The basic purpose of the NMF analysis was to decompose the data matrix into metacomponents and to determine their potential probabilities of contribution to the underlying variability structure. For example, as shown in **Table 5**, overall scores were used to break down the probability of each HMO that contributed to the observed pattern in the data with higher numbers having a higher probability of contribution (i.e., 2′-fucosyllactose contributed the most to the overall observed variability). Further separation of the analysis, by dividing the data into subgroups (such as population or BMI range), allowed for analysis of how these same components contributed to specified subsets of the data. Several detectable patterns were apparent when the NMF was used to analyze subgroups of the data. The extraction of HMO components with the highest feature scores led to 6 compounds (2′-fucosyllactose, LNFP I, LNFP II, 6′-sialyllactose, DFLNT, and FDSLNH); NMF scores for these HMOs (Supplemental Figure 1, Table 5) suggested that they contributed differently to the overall structure of HMO profiles across populations. For instance, 2′-fucosyllactose appeared to be highly influential to the variability of HMO profiles in the groups in Ghana, GB_U_, GB_R_, ET_U_, and ET_R_ but was less important in most other cohorts. DFLNT appeared to have a similar pattern, but it was also important in Kenya. Another example was 6′-sialyllactose, which contributed substantially to HMO profiles in the ET_R_, US_W_, US_C_, and Peru cohorts but in the other cohorts. Contributions to HMO-profile variability also seemed to differ by maternal BMI, parity, and time postpartum (**Tables 6****–****8**) (see **Supplemental Figures** 2 and **3** for related basis maps). For example, 2′-fucosyllactose was relatively more important in healthy-weight and overweight women than in underweight women, whereas difucosyllacto-*N*-hexaose was more important to the data structure in underweight women. Difucosyllactose appeared to decrease in relative contribution as BMI increased; conversely, 6′-sialyllactose, FDSLNH, and DSLNH appeared to increase in contribution as BMI increased. LNFP III was more important to the overall data structure for primiparous women than for multiparous women, and DSLNH has the greatest contribution in milk that was collected between 20 and 46 d postpartum. FLNH and DFLHN both contributed to lower amounts of the data structure as women got older (**Table 9**) (see Supplemental Figure 2 for related basis maps).

**TABLE 5:** Overall and population-specific NMF scores for each HMO^1^

	Ethiopia		Gambia							United States		
HMO	Rural (*n* = 40)	Urban (*n* = 40)	Rural (*n* = 40)	Urban (*n* = 40)	Ghana (*n* = 40)	Kenya (*n* = 42)	Peru (*n* = 43)	Spain (*n* = 41)	Sweden (*n* = 24)	Washington (*n* = 41)	California (*n* = 19)	Overall
2′FL	0.43	0.30	0.39	0.41	0.43	0.45	0.98	0.63	0.68	0.60	0.62	1.00
3FL	0.26	0.13	0.19	0.42	0.42	0.44	0.46	0.58	0.36	0.55	0.60	0.50
LNnT	0.18	0.15	0.26	0.22	0.34	0.17	0.32	0.30	0.25	0.42	0.14	0.45
3′SL	0.19	0.08	0.27	0.14	0.09	0.14	0.17	0.33	0.20	0.07	0.26	0.19
DFLac	0.49	0.30	0.32	0.67	0.29	0.31	0.54	0.69	0.56	0.23	0.47	0.51
6′SL	0.45	0.49	0.36	0.18	0.46	0.28	0.40	0.33	0.13	0.16	0.74	0.70
LNT	0.34	0.29	0.48	0.22	0.65	0.22	0.44	0.23	0.33	0.29	0.40	0.55
LNFP I	0.44	0.30	0.42	0.43	0.60	0.61	0.66	0.52	0.53	0.59	0.52	0.79
LNFP II	0.34	0.36	0.51	0.39	0.32	0.49	0.57	0.46	0.65	0.31	0.47	0.63
LNFP III	0.22	0.18	0.16	0.20	0.34	0.61	0.15	0.13	0.39	0.37	0.33	0.42
LSTb	0.28	0.19	0.39	0.22	0.35	0.24	0.22	0.27	0.18	0.25	0.14	0.33
LSTc	0.34	0.23	0.23	0.17	0.21	0.17	0.21	0.32	0.06	0.14	0.55	0.46
DFLNT	0.44	0.28	0.38	0.72	0.46	0.72	0.74	0.85	0.56	0.59	0.71	0.72
LNH	0.25	0.35	0.39	0.19	0.19	0.30	0.41	0.43	0.51	0.28	0.20	0.22
DSLNT	0.15	0.16	0.19	0.20	0.32	0.23	0.23	0.28	0.14	0.22	0.25	0.23
FLNH	0.15	0.13	0.19	0.16	0.50	0.06	0.47	0.42	0.25	0.29	0.29	0.31
DFLNH	0.43	0.32	0.34	0.61	0.23	0.43	0.34	0.41	0.21	0.44	0.41	0.37
FDSLNH	0.47	0.45	0.48	0.52	0.36	0.60	0.55	0.64	0.56	0.39	0.42	0.63
DSLNH	0.54	0.24	0.50	0.28	0.41	0.46	0.35	0.61	0.09	0.18	0.14	0.52

1 NMF scores represent the probability of contribution to (and importance of) a specified HMO variable to the basis component. DFLac, difucosyllactose; DFLNH, difucosyllacto-*N*-hexaose; DFLNT, difucosyllacto-*N*-tetrose; DSLNH, disialyllacto-*N*-hexaose; DSLNT, disialyllacto-*N*-tetraose; FDSLNH, fucodisialyllacto-*N*-hexaose; FLNH, fucosyllacto-*N*-hexaose; HMO, human milk oligosaccharide; LNFP, lacto-*N*-fucopentaose; LNH, lacto-*N*-hexaose; LNnT, lacto-*N*-neotetraose; LNT, lacto-*N*-tetrose; LSTb, sialyl-lacto-*N*-tetraose b; LSTc, sialyl-lacto-*N*-tetraose c; NMF, nonnegative matrix factorization; 2′FL, 2′-fucosyllactose; 3FL, 3-fucosyllactose; 3′SL, 3′-sialyllactose; 6′SL, 6′-sialyllactose.

**TABLE 6:** NMF scores describing BMI for individual HMOs^1^

	BMI, kg/m^2^		
HMO	Underweight(<18.5; *n* = 22)	Healthy weight(18.5–24.9; *n* = 242)	Overweight(≥25; *n* = 139)
2′FL	0.48	1.00	0.64
3FL	0.43	0.42	0.32
LNnT	0.16	0.54	0.35
3′SL	0.19	0.16	0.22
DFLac	0.54	0.45	0.32
6′SL	0.34	0.62	0.69
LNT	0.35	0.44	0.55
LNFP I	0.56	0.75	0.60
LNFP II	0.50	0.59	0.52
LNFP III	0.42	0.42	0.50
LSTb	0.07	0.30	0.30
LSTc	0.27	0.48	0.43
DFLNT	0.69	0.70	0.73
LNH	0.18	0.25	0.18
DSLNT	0.26	0.24	0.13
FLNH	0.51	0.26	0.54
DFLNH	0.58	0.45	0.25
FDSLNH	0.41	0.63	0.62
DSLNH	0.28	0.48	0.61

1 NMF scores represent the probability of contribution to (and importance of) a specified HMO variable to the basis component. DFLac, difucosyllactose; DFLNH, difucosyllacto-*N*-hexaose; DFLNT, difucosyllacto-*N*-tetrose; DSLNH, disialyllacto-*N*-hexaose; DSLNT, disialyllacto-*N*-tetraose; FDSLNH, fucodisialyllacto-*N*-hexaose; FLNH, fucosyllacto-*N*-hexaose; HMO, human milk oligosaccharide; LNFP, lacto-*N*-fucopentaose; LNH, lacto-*N*-hexaose; LNnT, lacto-*N*-neotetraose; LNT, lacto-*N*-tetrose; LSTb, sialyl-lacto-*N*-tetraose b; LSTc, sialyl-lacto-*N*-tetraose c; NMF, nonnegative matrix factorization; 2′FL, 2′-fucosyllactose; 3FL, 3-fucosyllactose; 3′SL, 3′-sialyllactose; 6′SL, 6′-sialyllactose.

**TABLE 7:** NMF scores describing parity for individual HMOs^1^

	Parity, children, *n*		
HMO	1 (*n* = 159)	2 (*n* = 111)	≥3 (*n* = 250)
2′FL	0.99	0.97	0.65
3FL	0.47	0.35	0.43
LNnT	0.29	0.43	0.38
3′SL	0.18	0.20	0.12
DFLac	0.51	0.31	0.42
6′SL	0.65	0.64	0.54
LNT	0.46	0.51	0.45
LNFP I	0.78	0.76	0.70
LNFP II	0.68	0.45	0.56
LNFP III	0.55	0.38	0.21
LSTb	0.29	0.29	0.26
LSTc	0.42	0.54	0.41
DFLNT	0.75	0.66	0.69
LNH	0.22	0.27	0.17
DSLNT	0.16	0.25	0.27
FLNH	0.37	0.27	0.28
DFLNH	0.30	0.30	0.35
FDSLNH	0.63	0.55	0.55
DSLNH	0.51	0.41	0.55

1 NMF scores represent the probability of contribution to (and importance of) a specified HMO variable to the basis component. DFLac, difucosyllactose; DFLNH, difucosyllacto-*N*-hexaose; DFLNT, difucosyllacto-*N*-tetrose; DSLNH, disialyllacto-*N*-hexaose; DSLNT, disialyllacto-*N*-tetraose; FDSLNH, fucodisialyllacto-*N*-hexaose; FLNH, fucosyllacto-*N*-hexaose; HMO, human milk oligosaccharide; LNFP, lacto-*N*-fucopentaose; LNH, lacto-*N*-hexaose; LNnT, lacto-*N*-neotetraose; LNT, lacto-*N*-tetrose; LSTb, sialyl-lacto-*N*-tetraose b; LSTc, sialyl-lacto-*N*-tetraose c; NMF, nonnegative matrix factorization; 2′FL, 2′-fucosyllactose; 3FL, 3-fucosyllactose; 3′SL, 3′-sialyllactose; 6′SL, 6′-sialyllactose.

**TABLE 8:** NMF scores describing time-postpartum quartiles for individual HMOs^1^

	Time postpartum, d			
HMO	20–46	47–63	64–78	79–161
2′FL	0.80	0.84	0.65	0.74
3FL	0.45	0.33	0.39	0.39
LNnT	0.38	0.44	0.26	0.55
3′SL	0.27	0.20	0.25	0.21
DFLac	0.48	0.27	0.39	0.39
6′SL	0.68	0.48	0.59	0.35
LNT	0.40	0.41	0.53	0.41
LNFP I	0.62	0.73	0.73	0.63
LNFP II	0.52	0.77	0.50	0.58
LNFP III	0.34	0.49	0.28	0.21
LSTb	0.29	0.31	0.31	0.29
LSTc	0.39	0.32	0.24	0.37
DFLNT	0.75	0.75	0.70	0.65
LNH	0.22	0.25	0.20	0.26
DSLNT	0.31	0.37	0.24	0.24
FLNH	0.60	0.31	0.17	0.35
DFLNH	0.40	0.45	0.24	0.30
FDSLNH	0.62	0.66	0.58	0.66
DSLNH	0.64	0.40	0.42	0.22

1 NMF scores represent the probability of contribution to (and importance of) a specified HMO variable to the basis component. DFLac, difucosyllactose; DFLNH, difucosyllacto-*N*-hexaose; DFLNT, difucosyllacto-*N*-tetrose; DSLNH, disialyllacto-*N*-hexaose; DSLNT, disialyllacto-*N*-tetraose; FDSLNH, fucodisialyllacto-*N*-hexaose; FLNH, fucosyllacto-*N*-hexaose; HMO, human milk oligosaccharide; LNFP, lacto-*N*-fucopentaose; LNH, lacto-*N*-hexaose; LNnT, lacto-*N*-neotetraose; LNT, lacto-*N*-tetrose; LSTb, sialyl-lacto-*N*-tetraose b; LSTc, sialyl-lacto-*N*-tetraose c; NMF, nonnegative matrix factorization; 2′FL, 2′-fucosyllactose; 3FL, 3-fucosyllactose; 3′SL, 3′-sialyllactose; 6′SL, 6′-sialyllactose.

**TABLE 9:** NMF scores describing maternal age quartiles for individual HMOs^1^

	Maternal age, y			
HMO	15–22	23–27	28–32	33–46
2′FL	0.97	0.71	0.83	0.69
3FL	0.43	0.43	0.46	0.43
LNnT	0.55	0.23	0.35	0.36
3′SL	0.19	0.26	0.18	0.15
DFLac	0.46	0.36	0.40	0.56
6′SL	0.66	0.58	0.63	0.71
LNT	0.54	0.30	0.53	0.44
LNFP I	0.75	0.62	0.82	0.67
LNFP II	0.60	0.48	0.63	0.49
LNFP III	0.24	0.54	0.36	0.43
LSTb	0.39	0.24	0.35	0.28
LSTc	0.47	0.39	0.44	0.46
DFLNT	0.71	0.78	0.68	0.65
LNH	0.27	0.27	0.22	0.21
DSLNT	0.36	0.15	0.32	0.29
FLNH	0.28	0.33	0.51	0.43
DFLNH	0.29	0.34	0.51	0.49
FDSLNH	0.48	0.63	0.64	0.64
DSLNH	0.48	0.44	0.62	0.59

1 NMF scores represent the probability of contribution to (and importance of) a specified HMO variable to the basis component. DFLac, difucosyllactose; DFLNH, difucosyllacto-*N*-hexaose; DFLNT, difucosyllacto-*N*-tetrose; DSLNH, disialyllacto-*N*-hexaose; DSLNT, disialyllacto-*N*-tetraose; FDSLNH, fucodisialyllacto-*N*-hexaose; FLNH, fucosyllacto-*N*-hexaose; HMO, human milk oligosaccharide; LNFP, lacto-*N*-fucopentaose; LNH, lacto-*N*-hexaose; LNnT, lacto-*N*-neotetraose; LNT, lacto-*N*-tetrose; LSTb, sialyl-lacto-*N*-tetraose b; LSTc, sialyl-lacto-*N*-tetraose c; NMF, nonnegative matrix factorization; 2′FL, 2′-fucosyllactose; 3FL, 3-fucosyllactose; 3′SL, 3′-sialyllactose; 6′SL, 6′-sialyllactose.

## DISCUSSION

Results from this study support our a priori hypothesis that concentrations of individual oligosaccharides and groupings thereof vary geographically in milk that is produced by healthy women. Indeed, absolute concentrations of all HMOs except for LNFP I varied in the studied cohorts. Because we took great care to collect and analyze the samples in a similar manner, we conclude that these differences are not a result of methodologic variation. In some cases (e.g., LNnT in GB_R_ compared with GB_U_), differences occurred despite similar genetic backgrounds, thereby suggesting that environmental factors may be important. In other cases (e.g., 2′-fucosyllactose in the US_C_ compared with US_W_), differences occurred across populations despite similar anthropometric and reproductive backgrounds, thereby suggesting that genetics, epigenetics, or other undocumented factors (e.g., micronutrient intake) also likely play important roles.

An understanding of the genesis and implications of HMO variation is important because increasing literature has suggested that individual HMOs might have particular structure-specific effects on infant health and risk of disease. For example, Mexican infants who received milk with low concentrations of 2′-fucosyllactose (nonsecretor milk) had higher diarrhea incidence (44) than of those who consumed appreciably higher amounts of 2′-fucosyllactose. Higher concentrations of FUT2-dependent HMOs such as 2′-fucosyllactose were also correlated with lower risk of allergy at 2 and 5 y of age in infants with high hereditary allergy risk (45), and HMOs including 2′-fucosyllactose attenuated food allergy symptoms in a mouse model (46). Data from our current study revealed significant variations in secretor status and 2′-fucosyllactose concentrations across global locations. Most strikingly, and similar to the reported high percentage of secretors in Mexico (32), we also showed very high percentages of secretors in women in PE and the US_C_ who self-identified as Hispanic. We hypothesize that this difference has been driven by evolutionary pressures that have conferred 2′-fucosyllactose–related health benefits in these populations, at least in their historical locations and within long-term behavioral and environmental constructs.

Other HMO isoforms are also likely related to health and disease risk in particular situations. For instance, a lower total HMO concentration and a higher proportion of 3′-sialyllactose were correlated with higher HIV transmission in Zambian infants (33), and HIV infection in lactating women was correlated with differences in HMOs both in Zambia and South Africa (33, 47). Moreover, HMO compositions have been associated with infant mortality and morbidity in HIV-exposed uninfected infants in Zambia (48). As such, increased consumption of these HMOs might be particularly important in this high-risk condition. Alderete et al. (49) have also shown that concentrations of individual HMOs in mother’s milk were associated with infant weight as well as lean and fat body masses in a US cohort. Similarly, Charbonneau et al. (50) reported that milk that was produced by Malawian mothers who were nursing severely stunted infants had lower HMO concentrations than in milk that was produced by mothers who were breastfeeding healthy-weight infants. Together, these studies suggest that the variation in HMO composition may affect the recipient infant’s metabolic phenotype, which is likely mediated through the gastrointestinal microbiome.

Research from the Bode laboratory (51) has also indicated that consumption of higher amounts of DSLNT, which is a sialylated HMO, may have been protective against the development of necrotizing enterocolitis-like symptoms in a rodent model. In the current study, we showed that maternal weight and BMI were inversely correlated with DSLNT concentrations, which suggest that maternal factors may partially contribute to HMO composition. The NMF analysis also suggested that there were somewhat different patterns in maternal BMI categories. Clearly, whether maternal adiposity is causally related to milk DSLNT (or any other HMO) concentration or HMO profiles or, instead, is a proxy for other maternal and environmental variables could not be ascertained from the current study. In addition, we recognize that body weight and BMI are not good indicators of adiposity during the postpartum period (52) and that other more sophisticated methods (e.g., dual-energy X-ray absorptiometry) will be needed to investigate this relation more thoroughly.

Note that, except for mothers in the US_C_, the mean concentration of 3-fucosyllactose in milk that was produced by the Swedish mothers in the current study was >2 times that of milk that was produced by women in all other cohorts. This result is in contrast with the previous work of Erney et al. (32) who detected no 3-fucosyllactose in the milk of Swedish mothers. In addition, although 3-fucosyllactose varied in the populations we studied, it was not the most variable, as suggested by Erney et al. (32). Instead, FLNH and LNFP III were 2- and 3-times more variable than 3-fucosyllactose. It is possible that methodologic differences between studies might explain this discrepancy.

Our data also revealed correlations between individual HMOs, which suggest that there are common synthetic pathways. Some of these correlations were anticipated; for instance, there were positive correlations between 2′-fucosyllactose, difucosyllactose, and LNFP I, which are HMOs that are all α1-2-fucosylated and highly depend on FUT2 activity. Other associations, such as the positive correlation between LNT and LNnT, were surprising. We had anticipated that the terminal galactose is either attached in β1-3-linkage to derive type 1 chains (e.g., LNT) or attached in β1-4-linkage to derive type 2 chains (e.g., LNnT); however, this would have yielded a negative correlation between LNT and LNnT, which suggests that other factors determine and limit chain elongation. Future studies that include genomic and transcriptomic data sets will help delineate HMO biosynthetic pathways and unravel how the synthesis of different HMOs is controlled. Forthcoming studies should be designed to determine correlations between HMOs and other milk components including the diverse communities of bacteria that are known to be in human milk (53–55).

One of our secondary objectives was to compare and contrast HMO contents and profiles between ethnically similar (and likely genetically similar) populations who were living in different locations. In this regard, note that there were several differences between milk that was produced by women in GB_U_ and GB_R_ (both Mandinka) and between milk that was produced by women in ET_U_ and ET_R_ (both Sidama). This finding suggests that there may be some effect of a relatively recent migration on the composition of HMOs rather than all of the variation being related strictly to genetic factors or simple-to-measure anthropometric and demographic variables. However, note that, unlike in all other cohorts for which milk was preserved by cold storage, milk collected from women in ET_R_ was first chemically preserved. Although our unpublished data (MK McGuire, KA Lackey, June 2016) suggest that the preservation method does not influence our ability to accurately characterize microbial communities in human milk, additional studies should be conducted to verify this finding.

In conclusion, the current study presents foundational data on what can be considered normal with regard to the HMO composition of milk that is produced by relatively healthy women in different locations around the world. Future studies are needed to determine how the variation in HMO composition is related to maternal and infant health and to generate hypotheses on HMO structure-function relations that can be tested in preclinical and clinical studies. Our data also provide a solid, and relatively unique, foundation on which to assess the deviation from a normal milk composition when women are not healthy (e.g., with diabetes, mastitis, or HIV). Future studies concerning this topic should strive to include women from regions (e.g., Asia) that were not included in the current study and women who are not healthy or are nursing unhealthy infants. The identification of the variation of the normal HMO composition in healthy women is just the beginning of a broader attempt to understand how sociocultural, evolutionary, environmental, and genomic aspects affect human milk composition and, subsequently, infant health. We posit that there is likely no one-size-fits-all construct when it comes to human milk composition and, thus, infant nutrition. Instead, we hypothesize that human milk composition has likely evolved differentially in such a way as to optimally nourish infants who are born in various social, environmental, genetic, and behavioral contexts. Future studies should be designed in such a way to examine this possibility and to also test it experimentally.

## Footnotes

23 Abbreviations used: DFLNT, difucosyllacto-*N*-tetrose; DSLNH, disialyllacto-*N*-hexaose; DSLNT, disialyllacto-*N*-tetraose; ET_R_, rural Ethiopia; ET_U_, urban Ethiopia; FDSLNH, fucodisialyllacto-*N*-hexaose; FLNH, fucosyllacto-*N*-hexaose; *FUT2*, galactoside 2-α-l-fucosyltransferase 2; *FUT3*, galactoside 3/4-l-fucosyltransferase; GB_R_, rural Gambia; GB_U_, urban Gambia; HMO, human milk oligosaccharide; LNFP, lacto-*N*-fucopentaose; LNnT, lacto-*N*-neotetraose; LNT, lacto-*N*-tetrose; LSTb, sialyl-lacto-*N*-tetraose b; LSTc, sialyl-lacto-*N*-tetraose c; NMF, nonnegative matrix factorization; US_C_, United States–California (Hispanic); US_W_, United States–Washington.

## References

[1] Bode L Human milk oligosaccharides: every baby needs a sugar mama. Glycobiology 2012;22:1147–62. 22513036 10.1093/glycob/cws074 PMC3406618

[2] Coppa GV, Pierani P, Zampini L, Carloni I, Carlucci A, Gabrielli O Oligosaccharides in human milk during different phases of lactation. Acta Paediatr Suppl 1999;88:89–94. 10.1111/j.1651-2227.1999.tb01307.x 10569230

[3] Kunz C, Rodriguez-Palmero M, Koletzko B, Jensen R Nutritional and biochemical properties of human milk, part I: general aspects, proteins, and carbohydrates. Clin Perinatol 1999;26:307–33. 10394490

[4] Newburg DS, Shen Z, Warren CD Quantitative analysis of human milk oligosaccharides by capillary electrophoresis. Adv Exp Med Biol 2000;478:381–2. 11065092 10.1007/0-306-46830-1_37

[5] Chaturvedi P, Warren CD, Altaye M, Morrow AL, Ruiz-Palacios G, Pickering LK, Newburg DS Fucosylated human milk oligosaccharides vary between individuals and over the course of lactation. Glycobiology 2001;11:365–72. 11425797 10.1093/glycob/11.5.365

[6] Davidson B, Meinzen-Derr JK, Wagner CL, Newburg DS, Morrow AL Fucosylated oligosaccharides in human milk in relation to gestational age and stage of lactation. Adv Exp Med Biol 2004;554:427–30. 15384616 10.1007/978-1-4757-4242-8_56

[7] Bao Y, Zhu L, Newburg DS Simultaneous quantification of sialyloligosaccharides from human milk by capillary electrophoresis. Anal Biochem 2007;370:206–14. 17761135 10.1016/j.ab.2007.07.004 PMC2441650

[8] Gabrielli O, Zampini L, Galeazzi T, Padella L, Santoro L, Peila C, Giuliani F, Bertino E, Fabris C, Coppa GV Preterm milk oligosaccharides during the first month of lactation. Pediatrics 2011;128:e1520–31. 22123889 10.1542/peds.2011-1206

[9] Gopal PK, Gill HS Oligosaccharides and glycoconjugates in bovine milk and colostrum. Br J Nutr 2000;84(Suppl 1):S69–74. 11242449 10.1017/s0007114500002270

[10] Tao N, DePeters EJ, German JB, Grimm R, Lebrilla CB Variations in bovine milk oligosaccharides during early and middle lactation stages analyzed by high-performance liquid chromatography-chip/mass spectrometry. J Dairy Sci 2009;92:2991–3001. 19528576 10.3168/jds.2008-1642

[11] Tao N, DePeters EJ, Freeman S, German JB, Grimm R, Lebrilla CB Bovine milk glycome. J Dairy Sci 2008;91:3768–78. 18832198 10.3168/jds.2008-1305

[12] Dong X, Zhou S, Mechref Y LC-MS/MS analysis of permethylated free oligosaccharides and N-glycans derived from human, bovine, and goat milk samples. Electrophoresis 2016;37: 1532–48. 26959529 10.1002/elps.201500561 PMC4963982

[13] Newburg DS Oligosaccharides in human milk and bacterial colonization. J Pediatr Gastroenterol Nutr 2000;30(Suppl 2):S8–17. 10749396

[14] LoCascio RG, Ninonuevo MR, Freeman SL, Sela DA, Grimm R, Lebrilla CB, Mills DA, German JB Glycoprofiling of bifidobacterial consumption of human milk oligosaccharides demonstrates strain specific, preferential consumption of small chain glycans secreted in early human lactation. J Agric Food Chem 2007;55:8914–9. 17915960 10.1021/jf0710480

[15] Sela DA, Chapman J, Adeuya A, Kim JH, Chen F, Whitehead TR, Lapidus A, Rokhsar DS, Lebrilla CB, German JB, The genome sequence of *Bifidobacterium longum* subsp. *infantis* reveals adaptations for milk utilization within the infant microbiome. Proc Natl Acad Sci USA 2008;105:18964–9. 19033196 10.1073/pnas.0809584105 PMC2596198

[16] Marcobal A, Barboza M, Froehlich JW, Block DE, German JB, Lebrilla CB, Mills DA Consumption of human milk oligosaccharides by gut-related microbes. J Agric Food Chem 2010;58:5334–40. 20394371 10.1021/jf9044205 PMC2866150

[17] Asakuma S, Hatakeyama E, Urashima T, Yoshida E, Katayama T, Yamamoto K, Kumagai H, Ashida H, Hirose J, Kitaoka M Physiology of consumption of human milk oligosaccharides by infant gut-associated bifidobacteria. J Biol Chem 2011;286:34583–92. 21832085 10.1074/jbc.M111.248138 PMC3186357

[18] Ruiz-Palacios GM, Cervantes LE, Ramos P, Chavez-Munguia B, Newburg DS *Campylobacter jejuni* binds intestinal H(O) antigen (Fuc alpha 1, 2Gal beta 1, 4GlcNAc), and fucosyloligosaccharides of human milk inhibit its binding and infection. J Biol Chem 2003;278:14112–20. 12562767 10.1074/jbc.M207744200

[19] Jiang X, Huang P, Zhong W, Tan M, Farkas T, Morrow AL, Newburg DS, Ruiz-Palacios GM, Pickering LK Human milk contains elements that block binding of noroviruses to human histo-blood group antigens in saliva. J Infect Dis 2004;190:1850–9. 15499543 10.1086/425159

[20] Jantscher-Krenn E, Lauwaet T, Bliss LA, Reed SL, Gillin FD, Bode L Human milk oligosaccharides reduce *Entamoeba histolytica* attachment and cytotoxicity in vitro. Br J Nutr 2012;108:1839–46. 22264879 10.1017/S0007114511007392

[21] Lin AE, Autran CA, Espanola SD, Bode L, Nizet V Human milk oligosaccharides protect bladder epithelial cells against uropathogenic *Escherichia coli* invasion and cytotoxicity. J Infect Dis 2014;209:389–98. 23990566 10.1093/infdis/jit464 PMC3883170

[22] Manthey CF, Autran CA, Eckmann L, Bode L Human milk oligosaccharides protect against enteropathogenic *Escherichia coli* attachment in vitro and EPEC colonization in suckling mice. J Pediatr Gastroenterol Nutr 2014;58:165–8. 24048169 10.1097/MPG.0000000000000172 PMC8865036

[23] Gonia S, Tuepker M, Heisel T, Autran C, Bode L, Gale CA Human milk oligosaccharides inhibit *Candida albicans* invasion of human premature intestinal epithelial cells. J Nutr 2015;145:1992–8. 26180242 10.3945/jn.115.214940

[24] Eiwegger T, Stahl B, Schmitt J, Boehm G, Gerstmayr M, Pichler J, Dehlink E, Loibichler C, Urbanek R, Szépfalusi Z Human milk–derived oligosaccharides and plant-derived oligosaccharides stimulate cytokine production of cord blood T-cells in vitro. Pediatr Res 2004;56:536–40. 15295093 10.1203/01.PDR.0000139411.35619.B4

[25] Kunz C, Rudloff S Potential anti-inflammatory and anti-infectious effects of human milk oligosaccharides. Adv Exp Med Biol 2008;606:455–65. 18183941 10.1007/978-0-387-74087-4_18

[26] Eiwegger T, Stahl B, Haidl P, Schmitt J, Boehm G, Dehlink E, Urbanek R, Szépfalusi Z Prebiotic oligosaccharides: in vitro evidence for gastrointestinal epithelial transfer and immunomodulatory properties. Pediatr Allergy Immunol 2010;21:1179–88. 20444147 10.1111/j.1399-3038.2010.01062.x

[27] Vázquez E, Barranco A, Ramírez M, Gruart A, Delgado-García JM, Martínez-Lara E, Blanco S, Martín MJ, Castanys E, Buck R, Effects of a human milk oligosaccharide, 2′-fucosyllactose, on hippocampal long-term potentiation and learning capabilities in rodents. J Nutr Biochem 2015;26:455–65. 25662731 10.1016/j.jnutbio.2014.11.016

[28] Bode L, Jantscher-Krenn E Structure-function relationships of human milk oligosaccharides. Adv Nutr 2012;3:383S–91S. 22585916 10.3945/an.111.001404 PMC3649474

[29] Kobata A Structures and application of oligosaccharides in human milk. Proc Jpn Acad Ser B Phys Biol Sci 2010;86:731–47. 10.2183/pjab.86.731 PMC3066539 20689231

[30] Kumazaki T, Yoshida A Biochemical evidence that secretor gene, Se, is a structural gene encoding a specific fucosyltransferase. Proc Natl Acad Sci USA 1984;81:4193–7. 6588382 10.1073/pnas.81.13.4193 PMC345395

[31] Xu Z, Vo L, Macher BA Structure-function analysis of human α1,3-fucosyltransferase. Amino acids involved in acceptor substrate specificity. J Biol Chem 1996;271:8818–23. 8621520 10.1074/jbc.271.15.8818

[32] Erney RM, Malone WT, Skelding MB, Marcon AA, Kleman-Leyer KM, O’Ryan ML, Ruiz-Palacios G, Hilty MD, Pickering LK, Prieto PA Variability of human milk neutral oligosaccharides in a diverse population. J Pediatr Gastroenterol Nutr 2000;30:181–92. 10697138 10.1097/00005176-200002000-00016

[33] Bode L, Kuhn L, Kim HY, Hsiao L, Nissan C, Sinkala M, Kankasa C, Mwiya M, Thea DM, Aldrovandi GM Human milk oligosaccharide concentration and risk of postnatal transmission of HIV through breastfeeding. Am J Clin Nutr 2012;96:831–9. 22894939 10.3945/ajcn.112.039503 PMC3441110

[34] R Development Core Team. R: a language and environment for statistical computing [Internet]. Vienna (Austria): R Foundation for Statistical Computing; 2015 [cited 2016 Jul 8]. Available from: http://www.R-project.org/.

[35] de Mendiburu F Agricolae: statistical procedures for agricultural research [Internet]. R package version 1.2-3. 2015 [cited 2016 Jul 8]. Available from: http://CRAN.R-project.org/package=agricolae.

[36] Ogle, DH. NCStats: helper functions for statistics at Northland College. R package version 0.4.4. Ashland (WI); 2015.

[37] Benjamini Y, Hochberg Y Controlling the false discovery rate: a practical and powerful approach to multiple testing. J Royal Stat Soc Ser B 1995;57: 289–300.

[38] Oksanen J, Blanchet GF, Kindt R, Legendre P, Minchin PR, O’Hara RB, Simpson GL, Solymos P, Stevens MHH, Wagner H Vegan: community ecology package [Internet]. R package version 2.3-3. 2016 [cited 2016 Jul 8]. Available from: http://CRAN.R-project.org/package=vegan.

[39] Wei T Corrplot: visualization of a correlation matrix [Internet]. R package version 0.73. 2013 [cited 2016 Jul 8]. Available from: http://CRAN.R-project.org/package=corrplot.

[40] Wickham H. ggplot2: elegant graphics for data analysis. New York: Springer-Verlag; 2009.

[41] Gaujoux R, Seoighe C A flexible R package for nonnegative matrix factorization. BMC Bioinformatics 2010;11:367. 20598126 10.1186/1471-2105-11-367 PMC2912887

[42] Brunet JP, Tamayo P, Golub TR, Mesirov JP Metagenes and molecular pattern discovery using matrix factorization. Proc Natl Acad Sci USA 2004;101:4164–9. 15016911 10.1073/pnas.0308531101 PMC384712

[43] Warnes GR, Bolker B, Bonebakker L, Gentleman R, Huber W, Liaw A, Lumley T, Maechler M, Magnusson A, Moeller S, gplots: various R programming tools for plotting data [Internet]. R package version 3.0.1 [cited 2016 Sep 6]. Available from: https://CRAN.R-project.org/package=gplots.

[44] Morrow AL, Ruiz-Palacios GM, Altaye M, Jiang X, Guerrero ML, Meinzen-Derr JK, Farkas T, Chaturvedi P, Pickering LK, Newburg DS Human milk oligosaccharides are associated with protection against diarrhea in breast-fed infants. J Pediatr 2004;145:297–303. 15343178 10.1016/j.jpeds.2004.04.054

[45] Sprenger N, Odenwald H, Kukkonen AK, Kuitunen M, Savilahti E, Kunz C FUT2-dependent breast milk oligosaccharides and allergy at 2 and 5 years of age in infants with high hereditary allergy risk. Eur J Nutr 2016 Feb 24 (Epub ahead of print; DOI: 10.1007/s00394-016-1180-6). 10.1007/s00394-016-1180-6 26907090

[46] Castillo-Courtade L, Han S, Lee S, Mian FM, Buck R, Forsythe P Attenuation of food allergy symptoms following treatment with human milk oligosaccharides in a mouse model. Allergy 2015;70:1091–102. 25966668 10.1111/all.12650

[47] Van Niekerk E, Autran CA, Nel DG, Kirsten GF, Blaauw R, Bode L Human milk oligosaccharides differ between HIV-infected and HIV-uninfected mothers and are related to necrotizing enterocolitis incidence in their preterm very-low-birth-weight. J Nutr 2014;144:1227–33. 24919691 10.3945/jn.113.187799

[48] Kuhn L, Kim HY, Hsiao L, Nissan C, Kankasa C, Mwiya M, Thea DM, Aldrovandi GM, Bode L Oligosaccharide composition of breast milk influences survival of uninfected children born to HIV-infected mothers in Lusaka, Zambia. J Nutr 2015;145:66–72. 25527660 10.3945/jn.114.199794 PMC4264023

[49] Alderete TL, Autran C, Brekke BE, Knight R, Bode L, Goran MI, Fields DA Associations between human milk oligosaccharides and infant body composition in the first 6 mo of life. Am J Clin Nutr 2015;102:1381–8. 26511224 10.3945/ajcn.115.115451 PMC6546222

[50] Charbonneau MR, O’Donnell D, Blanton LV, Totten SM, Davis JC, Barratt MJ, Cheng J, Guruge J, Talcott M, Bain JR, Sialylated milk oligosaccharides promote microbiota-dependent growth in models of infant undernutrition. Cell 2016;164:859–71. 26898329 10.1016/j.cell.2016.01.024 PMC4793393

[51] Jantscher-Krenn E, Zherebtsov M, Nissan C, Goth K, Guner YS, Naidu N, Choudhury B, Grishin AV, Ford HR, Bode L The human milk oligosaccharide disialyllacto-N-tetraose prevents necrotising enterocolitis in neonatal rats. Gut 2012;61:1417–25. 22138535 10.1136/gutjnl-2011-301404 PMC3909680

[52] Domer MC, Beerman KA, Ahmadzadeh A, Dasgupta N, Williams JE, McGuire MA, McGuire MK Loss of body fat and associated decrease in leptin in early lactation are related to shorter duration of postpartum anovulation in healthy US women. J Hum Lact 2015;31:282–93. 25596410 10.1177/0890334414565794

[53] Hunt KM, Foster JA, Forney LJ, Schütte UM, Beck DL, Abdo Z, Fox LK, Williams JE, McGuire MK, McGuire MA Characterization of the diversity and temporal stability of bacterial communities in human milk. PLoS One 2011;6:e21313. 21695057 10.1371/journal.pone.0021313 PMC3117882

[54] Fernández L, Langa S, Martín V, Jiménez E, Martín R, Rodríguez JM The microbiota of human milk in healthy women. Cell Mol Biol 2013;59:31–42. 24200019

[55] Urbaniak C, Angelini M, Gloor GB, Reid G. Human milk microbiota profiles in relation to birthing method, gestation and infant gender. Microbiome 2016;4:1. 10.1186/s40168-015-0145-y PMC4702315 26739322

